# Poorer Prognosis of Primary Signet-Ring Cell Carcinoma of the Breast Compared with Mucinous Carcinoma

**DOI:** 10.1371/journal.pone.0162088

**Published:** 2016-09-01

**Authors:** Xiufeng Wu, Zhenzhen Zhang, Xin Li, Qingzhong Lin, Gang Chen, Jianping Lu, Yi Zeng, Dan Hu, Kai Huang, Zhiwu Lin, Jun Yan

**Affiliations:** 1 Department of Surgery, Fujian Provincial Tumor Hospital, Teaching Hospital of Fujian Medical University, Fuzhou 350014, Fujian, People’s Republic of China; 2 Department of Pathology, The First Affiliated Hospital, Fujian Medical University, Fuzhou 350014, Fujian, People’s Republic of China; 3 Fujian Institute of Hematology, Union Hospital, Fujian Medical University, Fuzhou 350014, Fujian, People’s Republic of China; 4 Department of Pathology, Fujian Provincial Tumor Hospital, Teaching Hospital of Fujian Medical University, Fuzhou 350014, Fujian, People’s Republic of China; 5 Department of Surgery, Fujian Provincial Hospital, Teaching Hospital of Fujian Medical University, Fuzhou 350014, Fujian, People’s Republic of China; 6 Department of General Surgery, Nanfang Hospital, Southern Medical University, Guangzhou, Guangdong, 510515, P.R. China; University of Texas MD Anderson Cancer Center, UNITED STATES

## Abstract

Primary signet-ring cell carcinoma (PSRCC) of the breast is a rare entity and classified under mucin producing tumors in the WHO classification. However, little is known about the clinicopathological characteristics and clinical outcomes of PSRCC as opposed to mucinous carcinoma. Eleven patients with PSRCC in our center from 1995 to 2010 were evaluated in this study, as compared to 50 cases of mucinous breast cancer (MBC) during the same period. The clinicopathologic features of PSRCC compared to MBC were assessed. Furthermore, overall survival (OS) and disease-free survival (DFS) were calculated at 5 years of follow up. Patients with PSRCC showed more frequent lymphatic metastasis, higher Ki67 labeling index and more advanced stage disease than that of MBC (P = 0.018, p = 0.023, P = 0.000, respectively), although there was no difference in age, tumor size, and ER, PR expression between PSRCC and MBC. In addition, PSRCC was associated with simultaneous vimentin upregulation and E-cadherin downregulation. The 5-year OS of PSRCC (54.5%) was significantly lower than that of MBC (88%) (P = 0.004). Similarly, the DFS of PSRCC was poorer than that of MBC significantly (5-year DFS: 27.3% vs. 80%, P = 0.000).

**Conclusions:**

Our results confirmed the more aggressive behavior of PSRCC compared to MBC. This tumor is frequently associated with more frequent lymphatic metastasis, higher Ki67 labeling index, more advanced stage disease as well as simultaneous vimentin upregulation and E-cadherin downregulation. Different management guidelines should be considered for the two types.

## Introduction

Primary signet-ring cell carcinoma (PSRCC) of the breast is a rare entity with an incidence of 2 to 4.5%of all breast carcinomas [1, 2]. Saphir first described PSRCC as a variety of mucinous carcinoma in 1941 [3]. Since then, a few cases of PSRCC of the breast, each on the basis of limited numbers of such cases, have been reported in the English literature. Until 2003, PSRCC of the breast was separated from both infiltrating ductal and lobular carcinomas and classified as a special type of ‘mucin-producing carcinomas’ by the World Health Organization (WHO) [4]. Indeed, PSRCC of the breast is microscopically characterized by large intracytoplasmic mucin compressing the nuclei toward one end of the cell, which resembles signet rings. Clinically, PSRCC of the breast is defined as the presence of more than 20% of the signet-ring-like malignant cells in the tumor [4–6]. The histological origin of PSRCC is from either infiltrating lobular carcinoma or invasive ductal carcinoma [7–9]. Although placed under the same subtype of carcinoma as mucinous breast cancer (MBC), different morphology and biologic behavior between these two types of mucin-producing carcinoma has been observed. PSRCC is characterized by numerous cells containing intracytoplasmic mucin and associated with aggressive clinical behavior, as opposed to extracellular mucin and favorable prognosis of MBC [1, 2, 10–15]. It is important to distinguish PSRCC from MBC, since it is believed that treatment options and the clinical outcome are different [2]. However, on account of its rarity, the head-to-head comparison of characteristics and prognosis between PSRCC and MBC has yet to be reported.

In the current study, 11 cases of PSRCC from our center that had been documented in a 15-year period, and 50 cases of MBC within the same period were reviewed. We determined the differences in clinicopathological parameters and clinical outcomes between PSRCC and MBC. This is, to our best knowledge, the first demonstration of the differences in clinicopathological characteristics and clinical outcomes between PSRCC and MBC.

## Materials and Methods

### Ethics Statement

This study was done in accordance with the Declaration of Helsinki and approved by the Ethics Committees of Fujian Provincial Tumor Hospital. Written informed consent was obtained from all patients the time they were diagnosed with breast cancer by core needle biopsy at our center.

### Patients with PSRCC and MBC

We selected all patients with PSRCC and MBC from our center between 1995 and 2010 to avoid selection bias. This is a retrospective comparative analysis of consecutive patients with PSRCC and MBC with stage I–III disease, as reported from surgical pathologic records from our center between 1995 and 2010. Authors had access to identifying information after data collection. Inclusion criteria for PSRCC: (1) Over 20% of the signet-ring cell carcinoma; (2) the origin of the tumor is from the breast; Metastasis from other sites of signet-ring cell carcinoma is excluded [16, 17]. MBC included pure mucinous breast carcinoma (PMBC) comprising >90% mucin and mixed mucinous breast carcinoma (MMBC) [12, 13]. Written informed consent was obtained in all cases. This study was done in accordance with the Declaration of Helsinki and approved by the Ethics Committees of Fujian Provincial Tumor Hospital. The clinicopathologic features including age, axillary lymph node stage, pathologic stage, status of hormone receptors, HER2 expression, and Ki67 labeling index, as well as the information of surgery and systemic therapy, were evaluated. Two specialized pathologists (Chen Gang, Lu Jianping) with extensive experience in breast pathology performed a pathologic slide review. Nuclear staining of greater than 1% of tumor cells was regarded as ER and PR positivity. Immunohistochemistry (IHC) was used for confirmation of HER2 status according to the 2014 ASCO/CAP updated guideline [18]. 0, 1+, and 2+ scores indicated no cells with membrane staining, <10% of cells membrane stained and >10% of cells with a low or medium membrane staining, respectively. IHC score of 0 to 1+ was considered HER2 negative. Fluorescence in situ hybridization (FISH) should be performed as a confirmatory test for IHC 2+ cases. IHC 3+ with uniform, intense membrane staining of more than 30% of tumor cells or gene amplification detected by FISH in case of IHC 2+ were defined as positive. For Ki67 labeling index, a percentage of more than 15% of positive IHC stained nuclei was considered to be positive. The identification of PSRCC and MBC was carried out according to the World Health Organization’s histologic grading criteria used for breast cancer [19]. Pathologic staging was performed on the basis of the American Joint Commission on Cancer (AJCC) for breast cancer [20]. A follow-up of all patients was carried out according to medical record or telephone consultation. The clinical outcomes in terms of overall survival (OS) and disease-free survival (DFS) were calculated at 5years of follow up. OS was carried out from the date of initial treatment until death or the cutoff date of June 31, 2015. DFS was conducted from the date of initial treatment until the first observation of disease recurrence. The clinicopathologic features and prognosis of PSRCC and MBC were evaluated.

### Immunohistochemistry

In order to identify the biological background that can result in the difference between PSRCC and MBC, we evaluated the status of Vimentin, and E-cadherin using immunohistochemical analysis. Primary antibody against Vimentin (790–2917, Roche, Ventana) and E-cadherin (NCH-38, DAKO, Denmark) was detected using the DAKO REAL EnVision Detection System. Immunostaining intensity of Vimentin and E-cadherin was estimated in three categories: 0 (0–10%), 1+ (11–25%), 2+ (26–50%), and 3+ (>51%), and a cutoff value of>25% was defined as over-expression.

### Statistical analysis

The statistical analysis has been carried out using a statistical software package (SPSS 16.0 SPSS Inc., Chicago, IL, USA). The clinical and biologic characteristics were compared between groups using Chi square test. The OS and DFS were calculated by Kaplan–Meier estimator and the difference between groups was assessed using the log-rank test. All statistical tests were two-sided, and a p value less than 0.05 was considered statistically significant.

## Results

Retrospective comparative analysis of 11 consecutive patients with PSRCC and 50 with MBC with stage I–III disease, as reported from surgical pathologic records from our center between 1995 and 2010, was conducted. No data about included patients were missing. Table 1 showed the clinicopathological characteristics of 11 patients with PSRCC and 50 patients with MBC. All patients were female. The median age at diagnosis was 54 years (ranging from 32 to 76 years) in patients with PSRCC and 47 years (ranging from 27 to 78 years) in patients with MBC. There was no difference in age between PSRCC and MBC (P = 0.647). Similarly, no differences were seen in tumor size, or ER, PR expression between PSRCC and MBC. However, PSRCC showed more frequent lymphatic metastasis and higher Ki67 labeling index than that of MBC (P = 0.018, p = 0.023, respectively). In addition, PSRCC presented with more advanced stage disease than MBC (P = 0.000). There was no significant difference in surgical managements, chemotherapy, endocrine therapy, HER2 therapy, and radiotherapy between the two groups.

**Table 1:** Characteristics of patients with PSRCC and MBC.

Characteristics	PSRCC (n = 11; %)	MBC (n = 50; %)	P
Age (years)			0.647
≦50	6 (54.5%)	31 (62%)	
>50	5 (45.5%)	19 (38%)	
Tumor size			0.058
T1	0	16 (32%)	
T2	9 (81.8%)	31 (62%)	
T3	2 (18.2%)	3 (6%)	
T4	0	0	
N Stage			0.018
N0	3 (27.3%)	38 (76%)	
N1	4 (36.4%)	7 (14.0%)	
N2	2 (18.2%)	3 (6%)	
N3	2 (18.2%)	2 (4%)	
TNM stage			0.000
I	0	37 (74%)	
II	7 (63.6%)	8 (16%)	
III	4 (36.4%)	5 (10%)	
ER status			0.780
Positive	9 (81.8%)	39 (78%)	
Negative	2 (18.2%)	11 (22%)	
PR status			0.153
Positive	10 (90.9%)	35 (70%)	
Negative	1 (9.1%)	15 (30%)	
HER2 status			0.002
Positive	5 (45.5%)	4 (8%)	
Negative	6 (54.5%)	46 (92%)	
Ki-67			0.023
<14%	1 (9.1%)	23 (46%)	
>14%	10 (90.9%)	27 (54%)	
Surgery			0.905
Mastectomy	10 (90.9%)	46 (92%)	
Lumpectomy	1 (9.1%)	4 (8%)	
Chemotherapy			0.586
Yes	9 (81.8%)	37 (74%)	
No	2 (18.2%)	13 (26%)	
Endocrine therapy			0.330
Yes	10 (90.9%)	39 (78%)	
No	1 (9.1%)	11 (22%)	
HER2 therapy			0.069
Yes	3 (27.3%)	4 (8%)	
No	8 (72.7%)	46 (92%)	
Radiotherapy			0.399
Yes	4 (36.4%)	12 (24%)	
No	7 (63.6%)	38 (76%)	

### Prognosis of PSRCC in comparison to MBC

**Fig 1:**
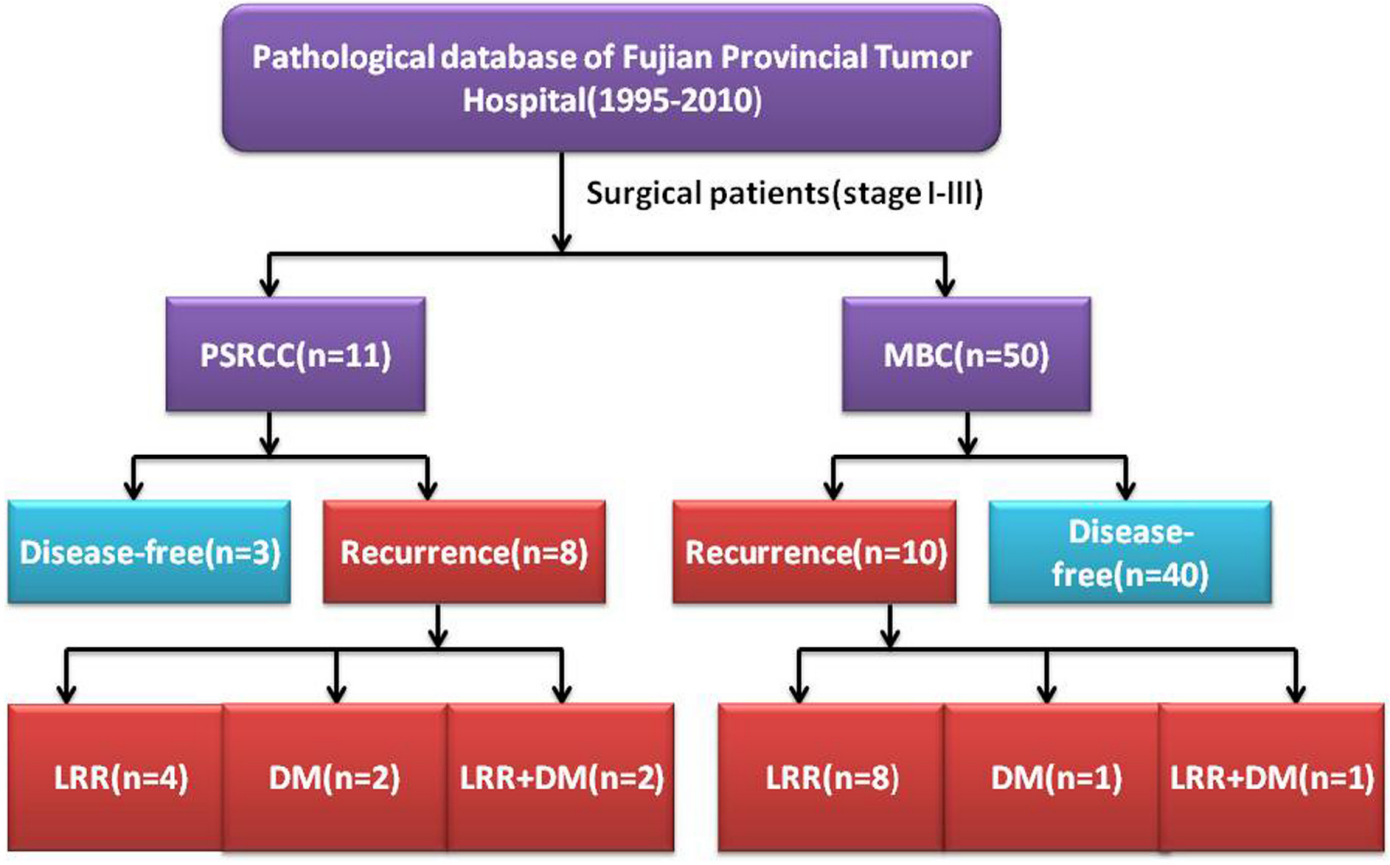
Patients with PSRCC and MBC in Fujian Provincial Tumor Hospital. PSRCC primary signet-ring cell carcinoma of the breast, MBC mucinous breast cancer, LRR locoregional recurrence, DM distant metastasis.

**Fig 2:**
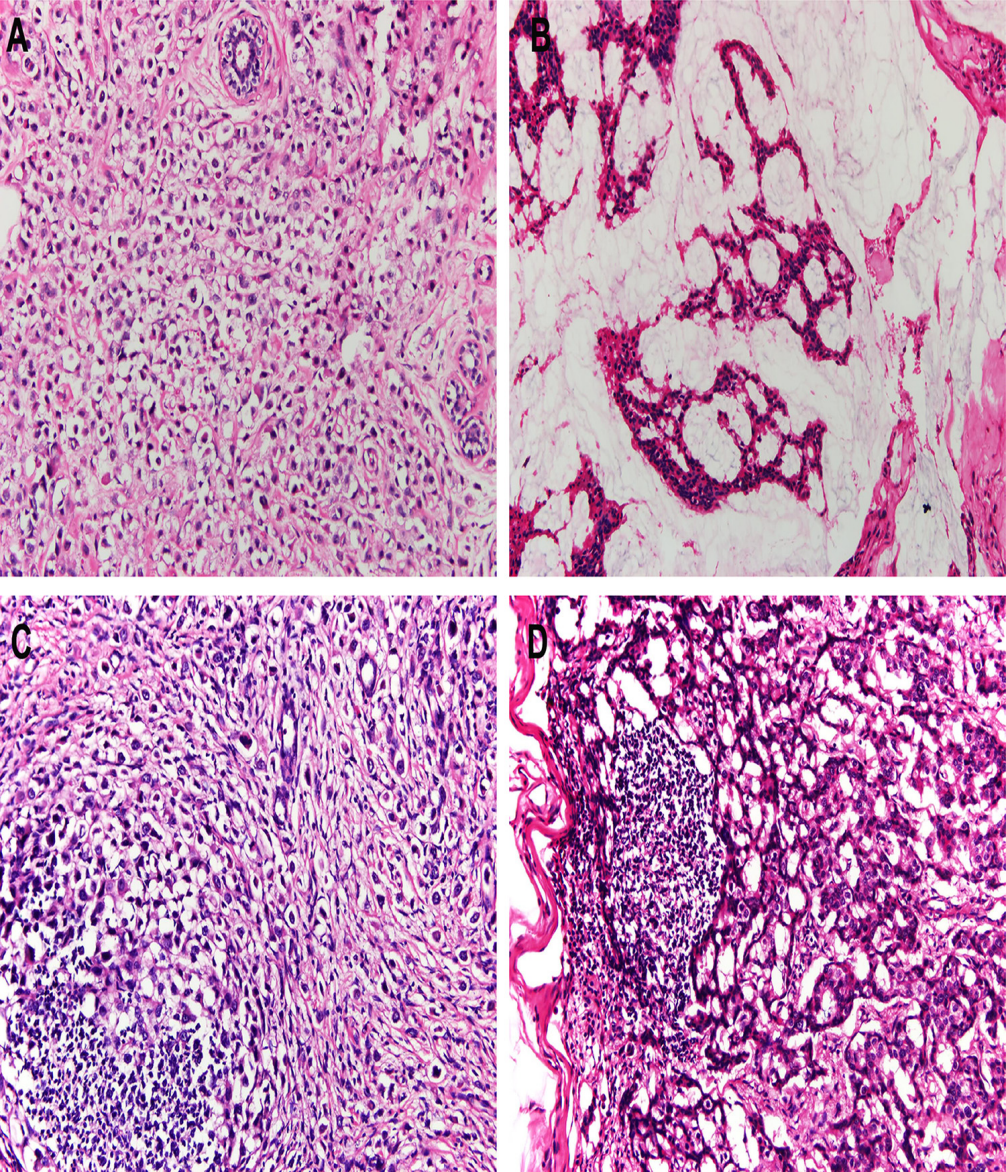
Histologic features of PSRCC and MBC. (A) Characteristic of PSRCC: The tumor was mainly composed of signet ring cells with large intracytoplasmic mucin compressing the nuclei toward the periphery of the cell. (B) Characteristic of MBC. The tumor was characterized by abundant extracellular mucin. (C) Lymph node metastases of PSRCC. (D) Lymph node metastases of MBC (Original magnifications 20x).

At the time of the last follow-up, no patients with PSRCC and MBC had been lost to follow-up. The median follow-up of PSRCC and MBC is 50 months (range from 4 to 114 months) and 51 months (range from 2 to 179 months), respectively. After a mean follow up period of 84 months, 8 (72.7%) patients with PSRCC suffered from recurrence (As shown in Fig 1). Among them, 4 patients experienced locoregional recurrence (LRR), 2 patients developed distant metastasis (DM), and 2 patients developed both LRR and DM. In contrast, a total of 10 (20%) patients with MBC suffered from recurrence, comprising 8 cases of LRR, 1 cases of DM, and 1 cases of both LRR and DM. Interestingly, morphologic features of PSRCC metastatic to lymph nodes was similar to that observed in the primary tumor with a signet-ring architecture (Fig 2). At the time of the last follow-up, 5 (41.4%) breast cancer related deaths were observed out of 11cases, as compared with 6 cases cancer deaths for patients with MBC. The 5-year OS of PSRCC was 54.5% as compared with 88% for patients with MBC (P = 0.004) (As shown in Table 2). Similarly, the DFS of PSRCC was poorer than that of MBC significantly (5-year DFS: 27.3 vs. 80%, P = 0.000), as shown in Table 2.

**Table 2:** Five-year OS and DFS of PSRCC versus MBC.

Age (years)	OS (%)	DFS (%)	P value
PSRCC	54.5% (6/11)	27.3% (3/11)	0.004
MBC	88% (44/50)	80% (40/50)	0.000

### Markers expression of Epithelial-Mesenchymal Transition (EMT)

**Fig 3:**
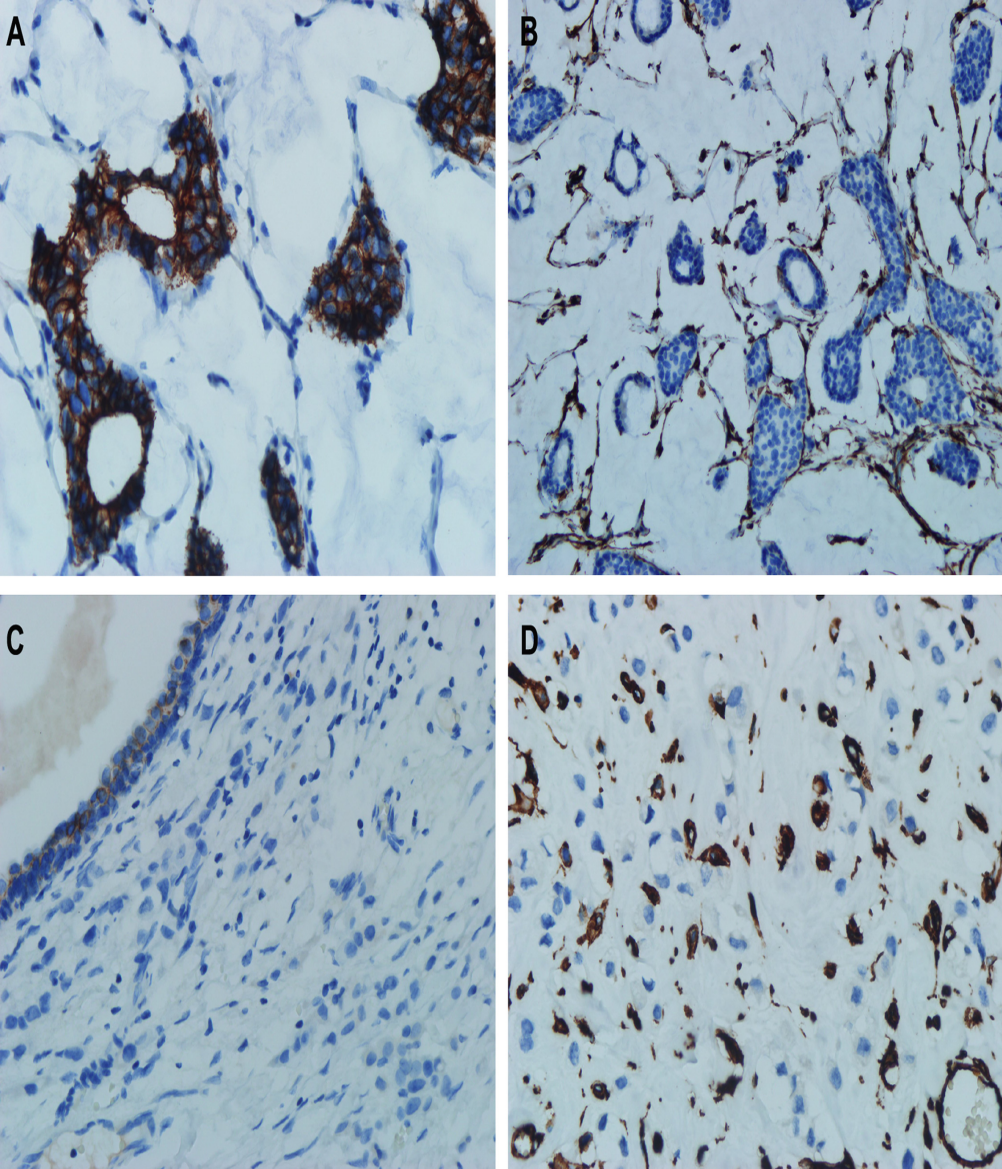
Representative immunostaining of Vimentin and E-cadherin in MBC and PSRCC. (A) MBC is positive for E-cadherin. (B) MBC is negative for Vimentin. (C) PSRCC is negative for E-cadherin. (D) PSRCC is positive for Vimentin (Original magnifications 40x).

Vimentin and E-cadherin are hallmarks of EMT, leading to motility [21–23]. Table 3 shows the Vimentin and E-cadherin expression for 11 patients with PSRCC as opposed to 50 patients with MBC by staining previously archived paraffin embedded sections with antibodies against vimentin and E-cadherin (Fig 3A & 3B). Vimentin expression in PSRCC was significantly higher than that in MBC (p = 0.000). However, E-cadherin expression in PSRCC was significantly lower than that in MBC (p = 0.000). Simultaneous vimentin upregulation and E-cadherin downregulation in PSRCC may induce EMT, enabling motility.

**Table 3:** Vimentin and E-cadherin expression in PSRCC and MBC.

	E-cadherin expression (%)	vimentin expression (%)	P value
PSRCC	27.3% (3/11)	72.7% (8/11)	0.000
MBC	92% (46/50)	10% (5/50)	0.000

## Discussion

PSRCC of the breast has been generally considered a rare entity originating from both invasive lobular and ductal carcinoma. For quite a long time, the clinicopathological characteristics and prognosis of PSRCC has been under-recognized in daily practice mainly because PSRCC has yet to be classified as a distinct entity. Indeed, PSRCC of the breast is frequently associated with lobular carcinoma, ductal carcinoma, or mucinous carcinoma. The diagnostic features of PSRCC is clinically established as the presence of more than 20 signet ring tumor cells per high power field (HPF) in any part of the tumor [12].

It has been concluded that majority of MBC is positive for hormone receptor, which is associated with better prognosis [13, 24]. Our results demonstrated a high percentage of hormone receptor expression in cases with PSRCC indicating a similar differentiation as MBC. Those findings were comparable to earlier studies [25].

As a subtype of mucin-producing carcinoma, the characteristics and clinical outcomes of PSRCC compared to those of MBC have not been reported yet. Our study found that there was no difference in ER expression, PR expression, age, and tumor size between PSRCC and MBC. However, patients with PSRCC were more likely to present with more advanced stages of disease and more frequent lymphatic metastasis at diagnosis than those with MBC (p = 0.000, p = 0.018, respectively), which was comparable to the findings in previous studies [1]. Along with the status of ER, PR, we also evaluated the expressions of HER2, Ki-67 labeling index in PSRCC. To the best of our knowledge, the HER2 status and Ki-67 labeling index have not been reported in the literature so far. Although there is no significant difference in HER2 expressions between PSRCC and MBC, patients with PSRCC presented with higher Ki-67 labeling index than MBC. There were 90.9% of PSRCCs over-expressed Ki-67 labeling index in the current study, which was associated with unfavorable prognosis [26]. Indeed, the Ki67 labeling index is also considered a predictive marker for response to chemotherapy [27–31]. Since patients with high Ki67 labeling index may derive much more benefit from adjuvant chemotherapy than those with low Ki67 labeling index [32], different treatment options based on the Ki-67 labeling index were suggested in the St. Gallen consensus 2009, especially for ER-positive breast cancers [33]. According to the St. Gallen consensus 2013 and 2015, high Ki67 labeling index was considered as a marker in support of chemotherapy for adjuvant treatment [34]. Our results demonstrated different treatment guidelines should be suggested for PSRCC and MBC due to different Ki67 labeling index between PSRCC and MBC.

The few series described in the literature indicate PSRCC had a more aggressive biological behavior and unfavorable prognosis than other forms of breast cancer without signet-ring cells. Merino et al. reported PSRCCs were frequently associated with poor prognosis, with 60% PSRCC-related death of 24 patients with PSRCC within 7 years [2]. Another retrospective study also demonstrated the increased axillary lymph nodal involvements and death rate in cases of signet-ring cell carcinoma than other forms of breast cancer without signet-ring cells [1]. Our study demonstrated that the DFS and OS are significantly shorter for PSRCC than that of MBC. The potential explanation for this result might be that PSRCC with large intracytoplasmic mucin compressing the nuclei toward the periphery of the cell may facilitate proliferation and progression of tumor cell. In contrast, MBC with large quantity of extracellular mucin may harbor scattered small foci of tumor cells. Previous study showed the process whereby epithelial cells acquire features of mesenchymal cells like higher migration, and invasion, is paralleled by a change in Vmentin and E-cadherin. In this study, simultaneous Vimentin upregulation and E-cadherin downregulation in PSRCC were evident, which may induce EMT in PSRCC. Thus, PSRCC associated with simultaneous vimentin upregulation and E-cadherin downregulation appears to be a biologically distinct subset that frequently shows unfavorable prognosis compared with MBC. However, the mechanism behind the aggressive behavior of PSRCC needs to be elucidated in future research. Consequently, due to significant difference in clinicopathological characteristics and prognosis between PSRCC and MBC, it is clinically important to separate PSRCC from MBC as a distinct type.

Since there were some limitations in this study in terms of a small study population, it is thus necessary to perform further research regarding the clinicopathological characteristics and prognosis of PSRCC in a large series with sufficient cases.

## Conclusions

This head-to-head retrospective comparative analysis confirms the more aggressive behavior of PSRCC compared to MBC. Patients with PSRCC were presented with higher Ki67 labeling index, more advanced stages of disease and more frequent lymphatic metastasis at diagnosis than those with MBC. In addition, we have shown that simultaneous vimentin upregulation and E-cadherin downregulation are associated with PSRCC and may induce EMT in PSRCC, resulting in aggressive behavior.

The discrimination of PSRCC from MBC is clinically important. Our results demonstrate that different treatment guidelines should be suggested for PSRCC and MBC. Surgical axillary staging by sentinel lymph node biopsy or axillary lymph node dissection should be considered for patients with PSRCC on account of its highly increased lymph node involvement. Even more aggressive postoperative therapy should be considered for patients with PSRCC. In contrast, surgical axillary staging is even not suggested for patients with MBC due to the favorable behavior of MBC [35].

## References

[1] Hull MT, Seo IS, Battersby JS, Csicsko JF. (1980) Signet-ring cell carcinoma of the breast. A clinicopathologic study of 24 cases. AM. J.Clin. Pathol 73:31–35. 6243441 10.1093/ajcp/73.1.31

[2] Merino MJ, Livolsi VA. (1981)Signet-ring carcinoma of the female breast: a clinicopathologic analysis of twenty-four cases. Cancer 48:1830–1837. 6269726 10.1002/1097-0142(19811015)48:8<1830::aid-cncr2820480821>3.0.co;2-h

[3] Saphir O. (1941) Mucinous carcinoma of the breast. Surg Gynecol Obstet72 9: 08–14.

[4] Ellis IO, Schnit SJ, Sastre-Garau X, Bussolati G, Tavassoli FA, Eusebi V, et al (2003) Invasive breast carcinoma In: Tavassoli FA, Devilee P, editors. Tumors of the breast and female genital system. Lyon: IARC P:30–48.

[5] Liu S-M, Chen D-R. (2000) Signet ring cell carcinoma of the breast. Case report. Pathol Int 50: 67–70. 10692181 10.1046/j.1440-1827.2000.01007.x

[6] Frost AR, Terahata S, Yeh IT, Siegel RS, Overmoyer B, Silverberg SG. (1995)The significance of signet ring cells in infiltrating lobular carcinoma of the breast. Arch Pathol Lab Med 119: 64–72. 7802556

[7] Fadare O. (2006) Pleomorphic lobular carcinoma in situ of the breast composed almost entirely of signet ring cells, Case report. Pathol Int.56: 683–690. 17040292 10.1111/j.1440-1827.2006.02030.x

[8] Qureshi SS, Shrikhande SV, Tanuja S, Shukla PJ. (2005) Breast metastases of gastric signet ring cell carcinoma: a differential diagnosis with primary breast signet ring cell carcinoma. J Postgrad Med 51: 125–132. 16006706

[9] Sato T, Muto I, Hasegawa M, Aono T, Okada T, Hasgawa J, et al (2007) Breast signet ring cell lobular carcinoma presenting with duodenal obsruction and acute pancreatitis. Asian J Surg.30: 220–223. 17638643 10.1016/s1015-9584(08)60026-3

[10] Sandhu J, Dubey VK, Makkar M, Suri V. (2013) Pure primary signet ring cell carcinoma breast: A rare cytological diagnosis. J Cytol 30: 204–6. 10.4103/0970-9371.117646 24130416 PMC3793361

[11] Karabagli P, Kilic H. (2013) Primary pure signet cell carcinoma of the breast: a case report and review of the literature. Breast Cancer 20:363–369. 10.1007/s12282-010-0210-0 20556557

[12] Di Saverio S1, Gutierrez J, Avisar E. (2008) A retrospective review with long term follow up of 11,400 cases of pure mucinous breast carcinoma. Breast Cancer Res Treat 111:541–7. 18026874 10.1007/s10549-007-9809-z

[13] Zhang M, Teng XD, Guo XX, Zhao JS, Li ZG. (2014) Clinicopathological characteristics and prognosis of mucinous breast carcinoma. J Cancer Res Clin Oncol. 140: 265–9. 10.1007/s00432-013-1559-1 24305754 PMC11823925

[14] Steinbrecher JS, Silverberg SG. (1976) Signet-ring cell carcinoma of the breast. The mucinous variant of infiltrating lobular carcinoma? Cancer. 37: 828–40. 175913 10.1002/1097-0142(197602)37:2<828::aid-cncr2820370231>3.0.co;2-n

[15] Harris M, Wells S, Vasudev KS. (1978) Primary signet ring cell carcinoma of the breast. Histopathology 2: 171–177. 208953 10.1111/j.1365-2559.1978.tb01707.x

[16] He CL, Chen P, Xia BL, Xiao Q, Cai FL. (2015) Breast metastasis of gastric signet-ring cell carcinoma: a case report and literature review. World J Surg Oncol. 13: 120–123. 10.1186/s12957-015-0538-1 25890325 PMC4386101

[17] Kim SJ. (2013) Magnetic resonance imaging features of inflammatory breast metastasis from gastric signet-ring cell carcinoma. Clin Imaging. 37: 569–73. 10.1016/j.clinimag.2012.09.005 23068056

[18] Wolff AC, Hammond ME, Hicks DG, Dowsett M, McShane LM, Allison KH, et al (2014) Recommendations for human epidermal growth factor receptor 2 testing in breast cancer: American Society of Clinical Oncology/College of American Pathologists clinical practice guideline update. Arch Pathol Lab Med 138:241–256. 10.5858/arpa.2013-0953-SA 24099077 PMC4086638

[19] Reis-Filho JS, Lakhani SR, Gobbi H. Metaplastic carcinoma In: Lakhani SR, Ellis IO, Schnitt SJ, et al (2012) Editors. WHO classification of tumours of the breast, 4th ed. Lyon: IARC P: 48–52.

[20] Edge SB, Byrd DR, Compton CC. (2009) American Joint Committee on cancer staging manual. 7th ed. New York: Springer.

[21] Kalluri R and Weinberg RA. The basics of epithelial-mesenchymal transition. J Clin Invest. 2009; 119(6):1420–1428. 10.1172/JCI39104 19487818 PMC2689101

[22] Polyak K and Weinberg RA. Transitions between epithelial and mesenchymal states: acquisition of malignant and stem cell traits. Nat Rev Cancer. 2009; 9(4):265–273. 10.1038/nrc2620 19262571

[23] Singh A and Settleman J. EMT, cancer stem cells and drug resistance: an emerging axis of evil in the war on cancer. Oncogene. 2010; 29(34):4741–4751. 10.1038/onc.2010.215 20531305 PMC3176718

[24] Jensen EV. (1966) Mechanism of estrogen action in relation to carcinogenesis. Proc Can Cancer Conf. 6: 143–208. 5973472

[25] Eltorky M, Hall JC, Osborne PT, el Zeky F. (1994) Signet-ring cell variant of invasive lobular carcinoma of the breast. A clinicopathologic study of 11 cases. Arch Pathol Lab Med. 118: 245–253. 8135627

[26] Wang GS, Zhu H, Bi SJ. (2012) Pathological features and prognosis of different molecular subtypes of breast cancer. Mol Med Rep 6: 779–861. 10.3892/mmr.2012.981 22797840

[27] Viale G, Regan MM, Mastropasqua MG, Maffini F, Maiorano E, Colleoni M, et al (2008) International Breast Cancer Study Group. Predictive value of tumor Ki-67 expression in two randomized trials of adjuvant chemoendocrine therapy for node-negative breast cancer. J Natl Cancer Inst 100: 207–219. 10.1093/jnci/djm289 18230798

[28] Aleskandarany MA, Rakha EA, Macmillan RD, Powe DG, Ellis IO, Green AR. (2011) MIB1/Ki67 labelling index can classify grade 2 breast cancer into two clinically distinct subgroups. Breast Cancer Res Treat.127: 591–9. 10.1007/s10549-010-1028-3 20623333

[29] Niikura N, Iwamoto T, Masuda S, Kumaki N, Xiaoyan T, Shirane M, et al (2012) Immunohistochemical Ki67 labeling index has similar proliferation predictive power to various gene signatures in breast cancer, Cancer Sci. 103: 1508–1520. 10.1111/j.1349-7006.2012.02319.x 22537114 PMC7659300

[30] von Minckwitz G, Schmitt WD, Loibl S, Müller BM, Blohmer JU, Sinn BV, et al (2013) Ki67 measured after neoadjuvant chemotherapy for primary breast cancer. Clin Cancer Res. 19: 4521–4552. 10.1158/1078-0432.CCR-12-3628 23812670

[31] Denkert C, Loibl S, Müller BM, Eidtmann H, Schmitt WD, Eiermann W, et al (2013) Ki67 levels as predictive and prognostic parameters in pretherapeutic breast cancer core biopsies: a translational investigation in the neoadjuvant GeparTrio trial. Ann Oncol 24: 2786–2879. 10.1093/annonc/mdt350 23970015

[32] Nitz U, Gluz O, Huober J, Kreipe HH, Kates RE, Hartmann A, et al (2014) Final analysis of the prospective SGAGO EC-Doc versus FEC phase III trial in intermediate-risk (pN1) early breast cancer: efficacy and predictive value of Ki67 expression. Ann Oncol 25: 1551–1558. 10.1093/annonc/mdu186 24827128

[33] Cuzick J, Dowsett M, Pineda S, Wale C, Salter J, Quinn E, et al (2011) Prognostic value of a combined estrogen receptor, progesterone receptor, Ki-67, and human epidermal growth factor receptor 2 immunohistochemical score and comparison with the Genomic Health recurrence score in early breast cancer. J Clin Oncol 29: 4273–4281. 10.1200/JCO.2010.31.2835 21990413

[34] Harbeck N, Thomssen C, Gnant M. (2013) St. Gallen 2013: brief preliminary summary of the consensus discussion. Breast Care (Basel) 8: 102–111. 24000280 10.1159/000351193 PMC3683952

[35] Paramo JC, Wilson C, Velarde D, Giraldo J, Poppiti RJ, Mesko TW. (2002) Pure mucinous carcinoma of the breast: is axillary staging necessary? Ann Surg Oncol 9: 161–164. 11888873 10.1007/BF02557368

